# Optimisation of a Novel Spiral-Inducing Bypass Graft Using Computational Fluid Dynamics

**DOI:** 10.1038/s41598-017-01930-x

**Published:** 2017-05-12

**Authors:** Andres Ruiz-Soler, Foad Kabinejadian, Mark A. Slevin, Paulo J. Bartolo, Amir Keshmiri

**Affiliations:** 1 Engineering and Materials Research Centre, Manchester Metropolitan University, Manchester, M1 5GD UK; 2 Department of Biomedical Engineering, University of Michigan, Ann Arbor, 48109-2110 USA; 3 Healthcare Science Research Centre, Manchester Metropolitan University, Manchester, M1 5GD UK; 4 School of Mechanical, Aerospace and Civil Engineering (MACE), The University of Manchester, Manchester, M13 9PL UK

## Abstract

Graft failure is currently a major concern for medical practitioners in treating Peripheral Vascular Disease (PVD) and Coronary Artery Disease (CAD). It is now widely accepted that unfavourable haemodynamic conditions play an essential role in the formation and development of intimal hyperplasia, which is the main cause of graft failure. This paper uses Computational Fluid Dynamics (CFD) to conduct a parametric study to enhance the design and performance of a novel prosthetic graft, which utilises internal ridge(s) to induce spiral flow. This design is primarily based on the identification of the blood flow as spiral in the whole arterial system and is believed to improve the graft longevity and patency rates at distal graft anastomoses. Four different design parameters were assessed in this work and the trailing edge orientation of the ridge was identified as the most important parameter to induce physiological swirling flow, while the height of the ridge also significantly contributed to the enhanced performance of this type of graft. Building on these conclusions, an enhanced configuration of spiral graft is proposed and compared against conventional and spiral grafts to reaffirm its potential benefits.

## Introduction

## Applications of Vascular Grafts

Arterial Bypass Graft (ABG) stenosis is currently a major concern for surgeons worldwide in treating coronary and peripheral arterial diseases. Each year over a million ABGs are performed, utilising either autologous vessels or prosthetic grafts commonly manufactured from Dacron (Polyethylene Terephthalate, PET) and expanded Polytetrafluoroethylene (ePTFE)^1^. Problems requiring the use of a graft include occluded vessels (i.e., stenosis), damaged vessels resulting from trauma or aneurysm, and the formation of a new tissue structure through regenerative therapies. Unfortunately, prosthetic grafts are known to exhibit unsatisfactory long-term performances^2^, whilst the most commonly used saphenous vein grafts fail on average between 10–15 years (5% failing within the first 30 days with no improvement over the last 40 years) due to size mismatch between graft and target vessels, turbulent flow, ischaemia and endothelial cell disruption and activation^3^.

Furthermore, the world is also facing a rapid rise in the incidence of diabetes mellitus and the need for haemodialysis. Similar to ABGs, stenosis at the graft-vein anastomosis is the major cause of failure of Arterio-Venous Grafts (AVGs) and have limited their use, resulting in arterio-venous fistula in the forearm to remain as an ideal vascular access for patients undergoing haemodialysis.

Much research is being performed to reduce failure rates and improve patency rates, particularly for vessels under 6 mm in diameter.

## Haemodynamic Effects of Spiral Flow

It is now widely accepted that haemodynamic factors play an important role in the formation and development of Intimal Hyperplasia (IH)^4, 5^ and acute thrombosis, which are the main causes of ABG and AVG failures. Several studies have shown a correlation between the haemodynamic factors and localised sites of intimal thickening, which in a conventional End-To-Side (ETS) configuration occur predominantly at the heel and toe of the anastomosis, on the arterial floor opposite the anastomosis, and on the suture line. Consequently, much research has been conducted in the past few decades to design grafts with longer patency, ideally longer than the life-span of the patient.

One of the most significant contributions to the improvement of haemodynamics in grafts was based on a research which showed that the ‘spiral flow’ is a natural phenomenon in the whole arterial system and is induced by the twisting of the left ventricle during contraction and then accentuated upon entering the aortic arch^6^. The benefit of this flow pattern lies in removing unfavourable haemodynamic environment such as turbulence, stagnation and oscillatory shear stress^7, 8^, which are believed to be the main causes of intimal hyperplasia at anastomotic configurations. Therefore, designs of new prosthetic grafts have started to take a new direction towards improving the patency of grafts through novel flow field augmentations. In this line, two of the most innovative designs are ‘SwirlGraft’ and ‘Spiral Flow Peripheral Vascular Graft’. The performance of ‘SwirlGraft’, developed by Caro and colleagues^9^ at Veryan Medical Ltd, is based on the swirling flow induced by the helical out-of-plane geometry and has reported less thrombosis development in animal experiments. ‘Spiral Flow Peripheral Vascular Graft’, initially studied by Stonebridge and colleagues^6, 7^ and subsequently commercialised by Vascular Flow Technologies (VFT) Ltd., works on the basis of the swirling flow induced by an internal ridge. The results of an early clinical non-randomised study for VFT peripheral bypass graft were promising and showed primary patency rates of 81% for above-the-knee bypasses and 57.3% for below-the-knee bypasses at 30 months of follow-up. The respective secondary patency rates were 81% and 64%^10^. In unpublished research, similar improvements were also found when using the spiral flow graft for AV access for haemodialysis (‘Spiral Flow AV Access Graft’).

A recent investigation by Kabinejadian *et al*.^11, 12^ has shown that graft out-of-plane helicity is more effective than a spiral ridge in inducing spiral flow, and their combination can further enhance the swirling effect in the flow. Whilst several researchers have numerically simulated the blood flow in out-of-plane graft geometries^13–18^, there are currently very few research papers in the literature investigating grafts with internal spiral ridge^19, 20^. Therefore, the focus of the present work will be restricted to this type of graft, with the aim of understanding the flow physics associated to the ridge design through conducting a parametric study on various geometrical parameters. The beneficiaries of this research would cover hospitals and the healthcare systems, medical practitioners and therapeutic technology industry, with a view to significantly reducing the incidence of early graft failure and thrombosis as well as improving long-term patency of grafts by controlling the laminar flow and slowing down intimal hyperplastic response.

## Method

### Computational Fluid Dynamics

In recent years, advances in vascular biology, biomechanics, medical imaging and computational techniques including Computational Fluid Dynamics (CFD) have provided the research community with a unique opportunity to analyse the progression of vascular diseases from a new angle and to improve the design of medical devices and develop new strategies for intervention. The increasing power-to-cost ratio of computers and the advent of methods for subject-specific modelling of cardiovascular mechanics have made CFD-based modelling sometimes even more reliable than methods based solely on *in vivo* or *in vitro* measurement^21^.

Generally, for prosthetic grafts, research is currently being undertaken under two separate strands; the first tends to focus on tissue-engineering and biomaterial science, while the second is concerned with biomechanics, flow field augmentations and haemodynamic forces. The attention of the present work is restricted to the latter and relies on CFD to investigate the effects of distal graft anastomosis configuration on secondary flow.

### Problem Definition

**Figure 1:**
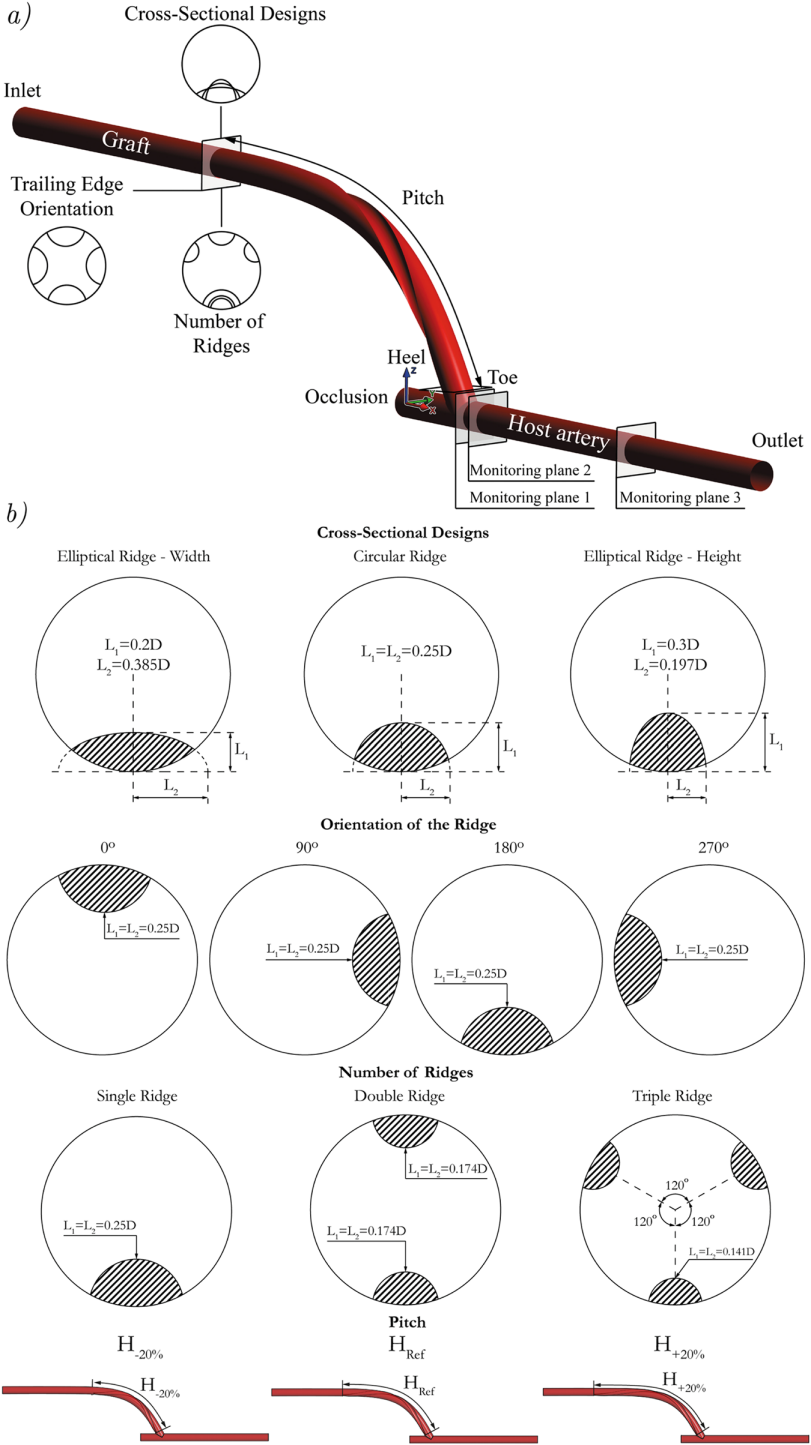
Schematics of (**a**) the computational model and (**b**) different ridge designs according to the dimensional specifications explained in Table 1.

In the present work, the design of a novel spiral-inducing graft has been assessed from the haemodynamic point of view. Figure 1a represents the computational model of an ETS distal graft anastomosis that connects the prosthetic graft and host artery with an anastomotic angle of 60 degrees. The internal diameters of the host artery and the graft are set to 6 mm. The swirling flow is induced by means of one or more spiral ridges in the internal wall of the graft. In order to understand the haemodynamic effects of the ridge design, different cross-sectional designs, positions, helical pitches and number of ridges have been tested, as indicated in Fig. 1b and Table 1. The appropriate comparison of the different designs of ridge(s) was achieved by setting the total cross-sectional area of ridge(s) to be constant (3.157 mm^2^), which homogenises the resistance against the flow, and consequently the pressure drop and boundary conditions amongst the different configurations tested here.

**Table 1:** Features of different configurations of ridge design and quantitative results in terms of pressure drop in the fluid domain, area-weighted average secondary velocity, spatial mean of wall shear stress in the host artery and percentage of cross-sectional area affected by recirculation.

	Cross-Sectional Designs			Trailing Edge Orientation				Number of Ridges			Pitch		
	*Elliptical Height*	*Circular*	*Elliptical Width*	*0°*	*90°*	*180°*	*270°*	*Single*	*Double*	*Triple*	*H*_*+20%*_	*H*_*ref*_	*H*_−*2*0%_
*L*_*1*_/*D*	0.3	0.25	0.2	0.25	0.25	0.25	0.25	0.25	0.174	0.141	0.25	0.25	0.25
*L*_*2*_/*D*	0.197	0.25	0.385	0.25	0.25	0.25	0.25	0.25	0.174	0.141	0.25	0.25	0.25
*Orientation of the ridge*(*s*)	180°	180°	180°	0°	90°	180°	270°	180°	0°, 180°	60°, 180°, 300°	180°	180°	180°
*Number of ridges*	1	1	1	1	1	1	1	1	2	3	1	1	1
*H*/*D*	14.42	14.42	14.42	14.42	14.42	14.42	14.42	14.42	14.42	14.42	17.31	14.42	11.54
*Mesh size ·10* ^*6*^ *Elements*	3.01	3.01	3.00	3.01	3.00	3.01	3.01	3.01	3.03	3.02	3.00	3.01	3.02
*Pressure drop [Pa]*	565.68	550.34	535.49	531.13	543.05	550.34	545.86	550.34	546.29	547.32	559.31	550.34	537.99
*V*_*yz*_ [*m s* ^−*1*^]*·10* ^−*3*^ *Plane 3*	5.95	5.01	5.41	9.37	9.58	5.01	22.00	5.01	6.87	11.46	5.24	5.01	6.45
*WSS* [*Pa*]	2.307	2.295	2.290	2.283	2.374	2.295	2.432	2.295	2.276	2.256	2.310	2.295	2.281
*Recirculation Plane 1*	7.38%	7.63%	7.78%	4.59%	6.25%	7.63%	5.31%	7.63%	6.70%	6.40%	7.47%	7.63%	7.56%
*Recirculation Plane 2*	8.71%	12.07%	13.51%	5.03%	3.82%	12.07%	3.52%	12.07%	10.83%	4.71%	11.35%	12.07%	11.76%

Four different geometrical parameters which are tested in the present work are described below.

#### Cross-Sectional Designs

The effect of the cross-sectional shape on haemodynamics has been assessed by considering circular and elliptical ridges. The circular ridge is considered as the reference case for further design parameters and the radius is set to 1.5 mm, giving a ratio of approximately 10% between the cross-sectional area of the ridge and graft (i.e., blockage ratio). The effects of the elliptical ridge have been studied through the variation of the vertical semi axis and keeping the cross-sectional area of the ridge fixed. As shown in Fig. 1b, in the numerical tests conducted here, the ratio between the vertical semi axis, L_1_, and the diameter of the graft, *D*, is set to 0.2 (elliptical ridge - width), 0.25 (circular ridge) and 0.3 (elliptical ridge - height).

#### Orientation of the Ridge

The procedure to analyse the impact of ridge’s trailing edge orientation on haemodynamics involves the rotation of the centre of the ridge to the positions 0°, 90°, 180° and 270°, along the circumference of the graft, near the anastomosis. For instance, the trailing edge orientations (TEOs) 0° and 180° represent cases where the ridge end at the toe and heel of the anastomosis, respectively. Note that the case of single circular ridge that was selected as the ‘reference’ configuration corresponds to the trailing edge orientation 180°, consistent with the current design of VFT’s ‘Spiral Flow Peripheral Vascular Graft’.

#### Number of Ridges

The effects of the number of ridges were assessed by means of three configurations with single, double and triple ridge. The single circular ridge is chosen as the reference cross-sectional shape to generate equally-spaced ridges. In the multi-ridge configurations, the radius of the circular ridges is decreased to keep the total cross-sectional area fixed at 3.157 mm^2^ for all three cases. Therefore, the radius of the ridge would be 1.042 mm and 0.844 mm in the double and triple ridge configurations, respectively.

#### Pitch of the Ridge

The influence of the pitch, H, on haemodynamics was evaluated through a variation of ± 20% in the helical pitch of the single circular ridge (*H*_*Ref*_ = 86.54 mm). In all cases, the orientation of the ridge was set as in the reference case (i.e., 180°).

### Computational Analysis

#### Computational Mesh

The computational domain used in this paper is based on finite-volume hybrid mesh consisting of prismatic elements for the near-wall and tetrahedral elements for the core regions and were generated using ANSYS-Meshing (Version 15.0). The number of elements varies between 3.00–3.03 million, depending on the geometry simulated in each case, in order to limit the variation in velocity and Wall Shear Stress to 1% with different mesh refinement levels. Skewness and orthogonal quality metrics were additionally checked to ensure the quality of the grids.

#### Governing Equations

The three-dimensional flow through the computational model is governed by the Navier-Stokes equations under the assumptions of laminar, stationary and isothermal:

Continuity equation,

1 $${\nabla }\cdot {\boldsymbol{u}}=0$$

Momentum equations,

2 $${\nabla }\cdot (\rho {\boldsymbol{uu}})=-{\nabla }p+{\nabla }\cdot (\mathop{\tau }\limits^{=})$$

3 $$\mathop{\tau }\limits^{{\boldsymbol{=}}}{\boldsymbol{=}}\mu {\boldsymbol{[}}{\boldsymbol{(}}{\rm{\nabla }}{\boldsymbol{u}}{\boldsymbol{+}}{\rm{\nabla }}{{\boldsymbol{u}}}^{{\bf{T}}}{\boldsymbol{)}}{\boldsymbol{-}}\frac{{\bf{2}}}{{\bf{3}}}{\rm{\nabla }}\cdot {\bf{u}}\mathop{{\bf{I}}}\limits^{{\boldsymbol{=}}}{\boldsymbol{]}}$$

where *ρ* is the density of the blood, ***u*** is the velocity vector, *p* is the static pressure, $\mathop{\tau }\limits^{=}$ is the stress tensor and *μ* is the dynamic viscosity. External body forces were neglected.

#### Non-Newtonian Flow

The blood has been characterised as an incompressible fluid with a density of 1050 kg m^−3^ ^22, 23^. The non-Newtonian behaviour of the blood has been implemented by means of the Carreau-Yasuda model^24^ that relates the dynamic viscosity, *μ*, as a function of the shear strain rate, $\dot{\gamma }$,

4 $$\mu ={\mu }_{\infty }+\frac{{\mu }_{0}-{\mu }_{\infty }}{{[1+{(\lambda \dot{\gamma })}^{a}]}^{\frac{1-n}{a}}}$$

where $\dot{\gamma }\,\,$represents a scalar measure of the rate of deformation tensor:

5 $$\dot{\gamma }=\sqrt{2tr({\mathop{D}\limits^{=}}^{2})}$$

where $\mathop{D}\limits^{=}=[{\nabla }{\boldsymbol{u}}+{({\nabla }{\boldsymbol{u}})}^{T}]/2$. The low-shear viscosity, high-shear viscosity, time constant, Yasuda exponent, and power law index are taken to be ${\mu }_{0}=22\cdot {10}^{-3}\,Pa\,s$, ${\mu }_{\infty }=2.2\cdot {10}^{-3}\,Pa\,s$, *λ* = 0.11*s*, *a* = 0.644, and *n* = 0.392, respectively^25^.

#### Boundary Conditions

A robust computational configuration of boundary conditions was implemented by considering a constant and uniform velocity of 0.317 m s^−1^ (*Re* = 570) at the inlet and zero pressure at the outlet. The wall has been considered as rigid^26, 27^ and no-slip boundary condition has been applied to all wall boundaries. The present computational model for spiral-inducing grafts was validated by running a series of steady-state simulations and compare against the experimental measurements of Kokkalis *et al*.^20^. One of the validation tests is shown in Supplementary Figure S1.

The governing equations of the fluid dynamic problem were solved using a high resolution scheme in ANSYS CFX (Version 15.0). The convergence of solutions was ensured by the global balance of the conservation equations and a RMS (Root Mean Squared) residual criterion of 10^−6^.

While most of the present simulations were conducted using steady-state condition, a series of transient simulations were also conducted to obtain more advanced haemodynamics parameters (discussed further below). For transient simulations, an implicit second-order backward Euler method was used for the transient scheme with a time step of 0.01 s. The residual criterion to ensure the convergence of the numerical method was set to 10^−6^. The results presented here were extracted from the last of four simulation periods in order to avoid initial instabilities of the numerical procedure.

### Optimisation Criteria

Although it is widely accepted that haemodynamic parameters play an important role in the patency rate of graft anastomosis, there is no unanimity about the optimal flow pattern that improves the efficiency of the grafting^28^. Several authors have highlighted the benefits of high Wall Shear Stress (WSS) for avoiding the formation of plaque^29, 30^, increase of intimal medial thickness^31^ and proliferation of fibroatheroma and intermediate lesion^32^. In contrast, others suggest that high values of WSS may result in endothelial lesions^33^. The paucity of *in vivo* data to support the existing hypotheses in the development of intimal hyperplasia and atherosclerosis in different bypass configurations is currently a major challenge^28^, which makes it difficult to find haemodynamically ‘optimum’ configurations.

Nevertheless, the swirling flow is considered to be a beneficial physiological mechanism to reduce abnormal flow conditions^34^ in order to prevent thrombosis, intimal hyperplasia and atherosclerotic lesions^20, 35, 36^ (i.e., the main causes of graft failure). Consequently, consistent with Kabinejadian *et al*.^11^, the haemodynamic ‘optimisation criteria’ of the spiral-inducing graft in the present study will be based on the assumptions of high wall shear stress, high secondary velocity and reduction of separation and recirculation zones, as potential favourable haemodynamic factors.

## Results

### Pressure Drop

The blood flow supply to organs and tissues is regulated by the resistance against the flow and the resulting pressure drop. Together with the rheology of the blood, the geometry of vessels are the main parameters involved in the adjustment of blood flow by means of vasoconstrictor and vasodilator mechanisms. In line with the above, the design strategy established in the present study is based on setting the total cross-sectional area of ridge(s) to be constant. This limits the difference in pressure drop of different cases to 3.5% with respect to the reference configuration (as shown in Table 1), thus, leading to a numerically valid comparison between different design parameters.

### Secondary Velocity

The swirling motion has been characterised through the secondary velocity contours and crossflow streamlines at the monitoring plane 3, which is located 50 mm distal from the toe of the anastomosis where the effect of graft designs and the swirling motion are more meaningful. The secondary velocity was defined as the composition of the velocities *v* and *w* along the *Y* and *Z* axes in the host artery, respectively.

6 $${V}_{yz}=\sqrt{{v}^{2}+{w}^{2}}$$

**Figure 2:**
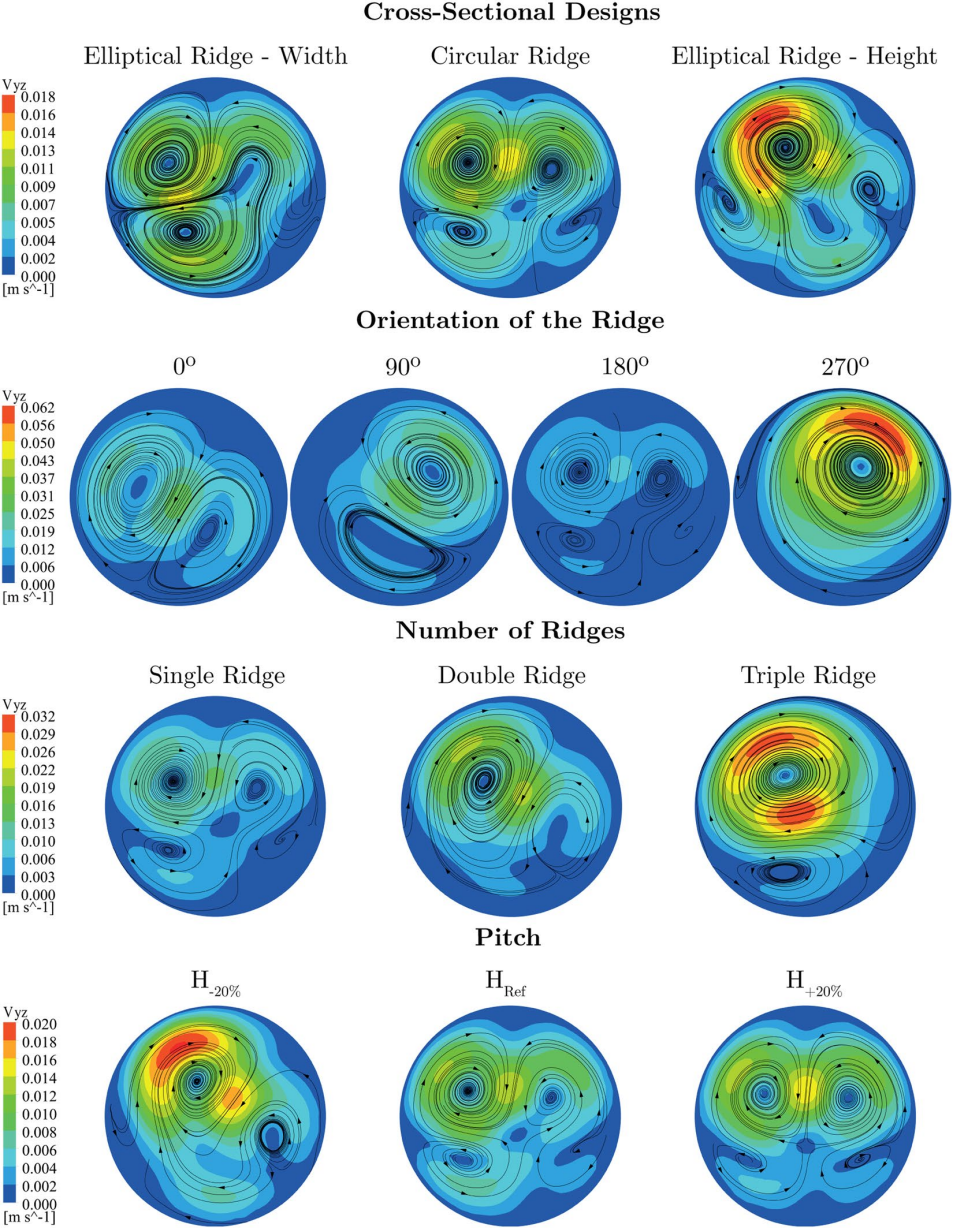
Contours of secondary velocity and crossflow streamlines for different ridge designs at monitoring plane 3.

Note that the direction of the flow in all secondary velocity distributions represented in Fig. 2 should be interpreted as into the page.

In terms of the cross-sectional shape, both elliptical ridge configurations show enhanced average secondary magnitudes with respect to the circular configuration, increasing the improvement by the height of the elliptical ridge to 19%.

The helical flow generated by different orientations of the ridge results in a number of prominent flow features worth highlighting. The trailing edge orientation 270° stands out amongst the other configurations by reaching the highest secondary velocity and producing a single dominant spiral as a result of dissipation of the weaker vortices. Although one would assume that the trailing edge orientations 90° and 270° should produce similar distributions in the present case, the results highlight significant differences, mainly due to the spiral direction along the pitch. Since the secondary flow in the distal section of the graft is clockwise, in the case of trailing edge orientation 90°, the induced rotation by the ridge is supressed by the near sidewall effect in the host artery upon impingement on the arterial bed, as illustrated in Supplementary Figure S2. However, in the case of trailing edge orientation 270°, the swirling flow receives a lesser degree of suppression from the surrounding wall boundaries. This phenomenon could also be observed at plane 3 where the average secondary velocity magnitude in the 270° model is over 4 times higher than that produced by the reference case (180°). Although each model shows a different secondary velocity distribution, the 180° and 270° orientations show the weakest and strongest average secondary velocity magnitudes, respectively.

The effects of increasing the number of ridges results in an increase in average secondary velocity magnitude of 37% when comparing the double ridge configuration with the reference case. The triple ridge design with three equally-spaced ridges at 60°, 180° and 300° leads to an even more significant increase of secondary velocity magnitude and shows the implicit effect of the trailing edge orientation through the ridges located at the orientations 60° and 300°. A further study using double ridge with different trailing edge orientations (90° and 270° instead of 0° and 180°) revealed the influence of the orientation over the number of ridges, as shown in Supplementary Figure S3.

The influence of the pitch is evidenced by a noteworthy increase of approximately 30% in secondary velocity magnitude by decreasing the pitch.

### Wall Shear Stress

The WSS characterises the tangential fluid forces that act on the vessel wall. As was alluded to earlier, the intimal thickening and restenosis due to intimal hyperplasia is normally characterised by low WSS^29–32^. From the haemodynamic point of view, fibrinogen/fibrin is normally deposited at low shear rates and at areas exposed to eddies, flow separations and stasis (i.e., after stenosis and after areas of flow disturbance), thus, making the WSS metric even more relevant in the present study^11^.

**Figure 3:**
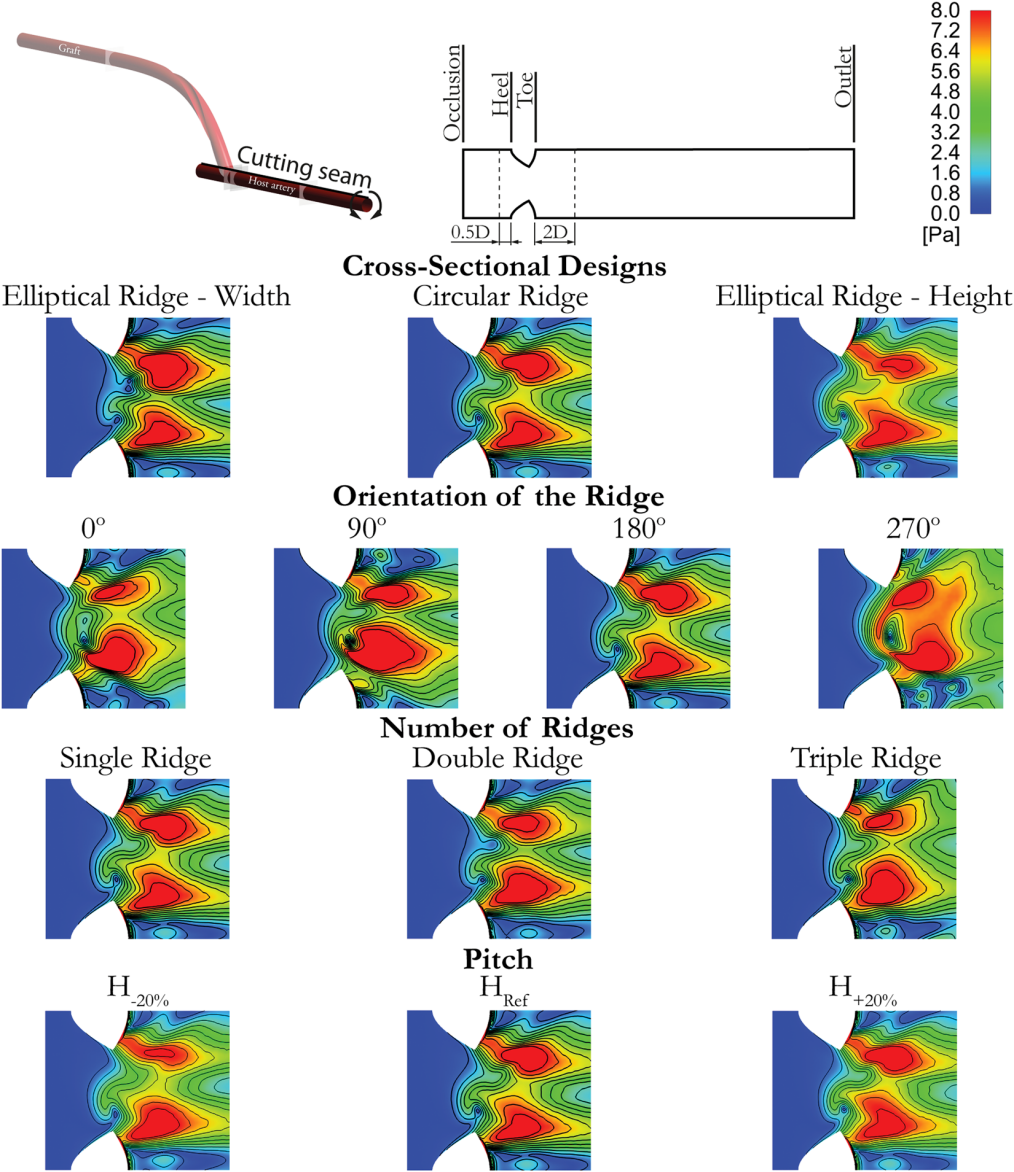
Distributions of WSS onto the developed surface of the host artery for different ridge designs.

Figure 3 presents the distributions of WSS on an unfolded model of the host artery, which has been opened ventrally and the direction of the flow is from left to right. The spatial means of such distributions in the host artery are given in Table 1.

The increase of the ridge height generates an increase of WSS on the bed in magnitude and asymmetry, slightly shifting the peak regions to the lateral side of the host artery.

The trailing edge orientation again represents an important parameter since the most homogeneous distribution and the highest average WSS are obtained by the 270° configuration; this trailing edge orientation also results in the high-shear region further extending proximally towards the occluded section of the host artery. Moreover, in agreement with the results presented in Fig. 2, the 90° configuration also results in higher average WSS magnitude compared to 0° and 180° configurations.

The increase in the number of ridges results in consecutive and slight decreases of the spatial mean WSS, while the degree of asymmetry in the distributions tend to increase with respect to the reference case.

The variation of the ridge pitch results in minor effects on the spatial mean of WSS in the host artery, returning differences less than 1% as shown in Table 1.

### Flow Separation and Recirculation

Abnormal flow conditions and recirculation are associated to regions of low WSS that lead to cholesterol deposition, atheroma growth thrombus formation and, eventually, IH development^5^. Therefore, the focus of this section is on the recirculation and retrograde flow regions.

**Figure 4:**
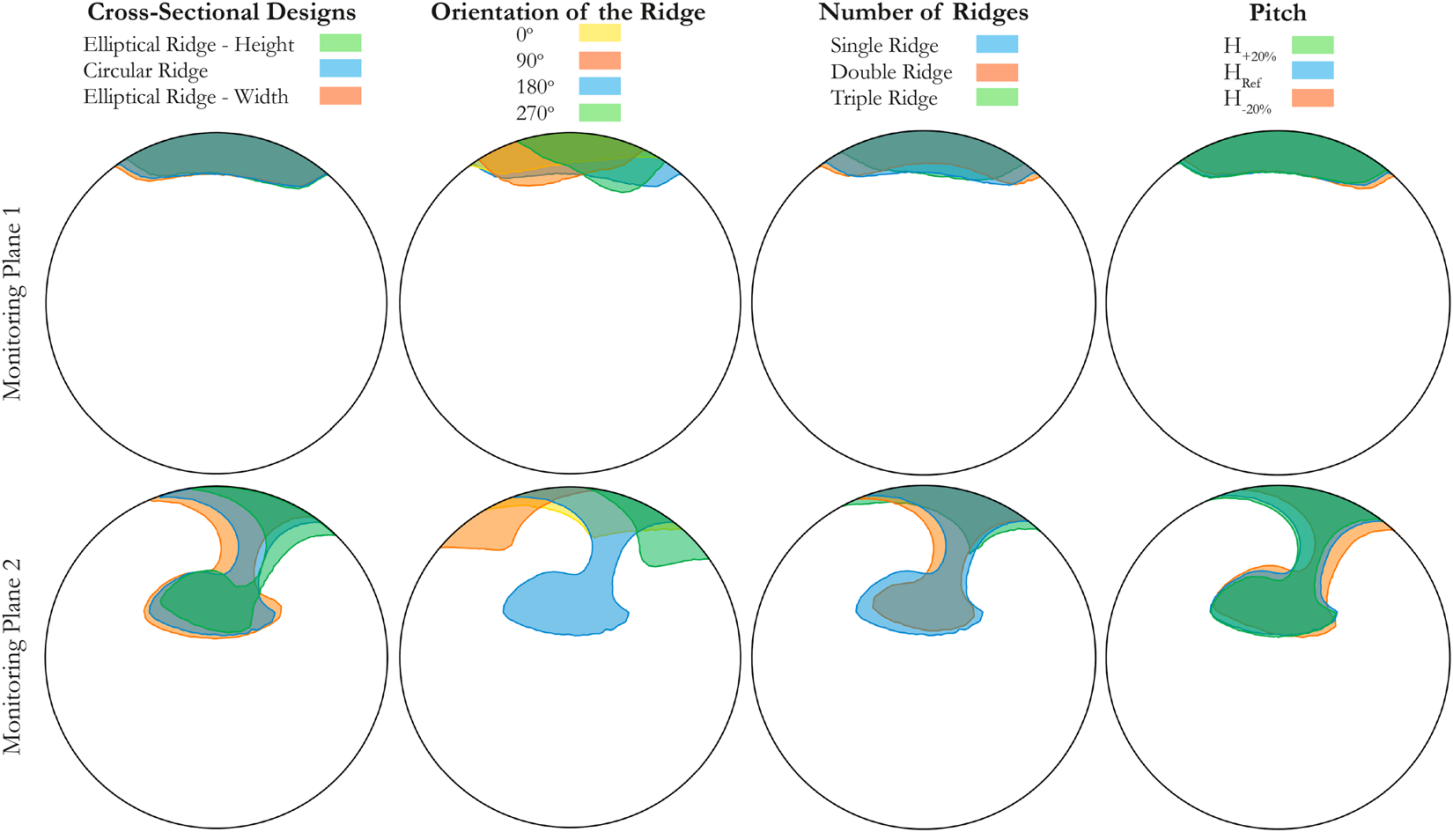
Regions of retrograde flow with different designs at monitoring planes 1 and 2 (monitoring plane 3 is free of recirculation).

Figure 4 shows the regions of retrograde flow (negative axial velocity) generated by different graft designs at monitoring planes 1 and 2 located at 1 mm and 5 mm distal from the toe of the anastomosis, respectively. These regions were insignificant at plane 3, hence not included in Fig. 4. The direction of the flow should be interpreted as into the page. The percentage of cross-sectional area of the host artery affected by this adverse effect is given in Table 1.

In all cases, the recirculation zones are found at the toe side of the host artery close to the anastomosis. At monitoring plane 1, except for the trailing edge orientation, the other three design parameters present minor effects on the flow separation zones with the maximum 1.2% difference compared to the reference case. The variation of the ridge trailing edge orientation with respect to the reference case, however, results in a slight decrease of the flow separation zone but an increase in the degree of asymmetry in these regions.

Very distinct recirculation zones are found at monitoring plane 2, most of which can be seen to extend towards the centre of the lumen and to rotate due to the spiral motion of the graft outlet flow impinging onto the host arterial bed. The increase of the ridge height and width tend to decrease (3.4%) and increase (1.4%) the recirculation region, respectively. The variation of the trailing edge position with respect to the reference model results in significant reduction in both retrograde flow area (e.g. 8.6% for trailing edge orientation 270°) and the extent of recirculation region towards the centre of the lumen. Another interesting point to highlight here is how the recirculation regions of the orientations 270° and 90° rotate clockwise and anticlockwise, respectively, around the axis of the host artery. This can be explained again by considering the interaction between the induced rotations by the ridge and surrounding wall boundaries.

Increasing the number of ridges within the graft leads to reduction of the disturbed zones. In particular, the results of the triple ridge design are of significant interest here, where 7.4% reduction is achieved.

Finally, similar to the results obtained in terms of WSS, the modification of the pitch leads to insignificant effects on the variation of recirculation regions.

## Discussion

### Spiral-Inducing Bypass Grafts

The recognition of the physiological blood flow as spiral in the whole arterial system has led to novel designs of spiral-inducing prosthetic graft including Spiral Laminar Flow (SLF) peripheral vascular graft commercialised by Vascular Flow Technologies (VFT) Ltd., a design which is the main focus of the present work. Through inducing spiral blood flow using an internal ridge, the SLF graft is claimed to: (1) reduce laterally directed forces and near-wall turbulence, (2) suppress acute thrombus formation with no increase in platelet activation, (3) reduce WSS gradient and Oscillatory Shear Index (OSI), (4) enhance oxygen flux to the arterial wall and (5) reduce luminal surface low-density lipoproteins concentration^10, 20^.

While the initial results^10^ reported in the literature highlight the potential for the idea of spiral-inducing grafts in bypass surgeries, in a very recent clinical investigation, Bechara *at al*.^37^ carried out a single-centre study on patients undergoing infrainguinal bypass using VFT’s spiral laminar flow graft against Propaten (a heparin-bonded ePTFE graft produced by W.L. Gore) and found that the spiral laminar flow graft had not led to higher patency rates in comparison to the conventional ePTFE grafts. The numerical simulations of Kabinejadian *et al*.^11^ also found that graft out-of-plane helicity is significantly more effective than a single spiral ridge.

### Design Parameters and Clinical Relevance

The above findings highlight the need for further investigation on the design and improving the performance of these grafts with more effective spiral designs, which is the subject of the present work. Therefore, building on the important role of the haemodynamic parameters and the recognised sensitivity of the flow pattern to the geometry^11, 38–40^, the present investigation has assessed different designs of a peripheral prosthetic graft with internal spiral ridge(s).

The design condition of constant total cross-sectional area adopted here results in the same level of resistance against the flow and consequence pressure drop, enabling the appropriate comparison of different configurations. Based on the widely accepted assumptions postulated earlier, the flow patterns have been studied in order to increase the swirling flow and wall shear stress as well as reducing flow separation and recirculation zones.

**Figure 5:**
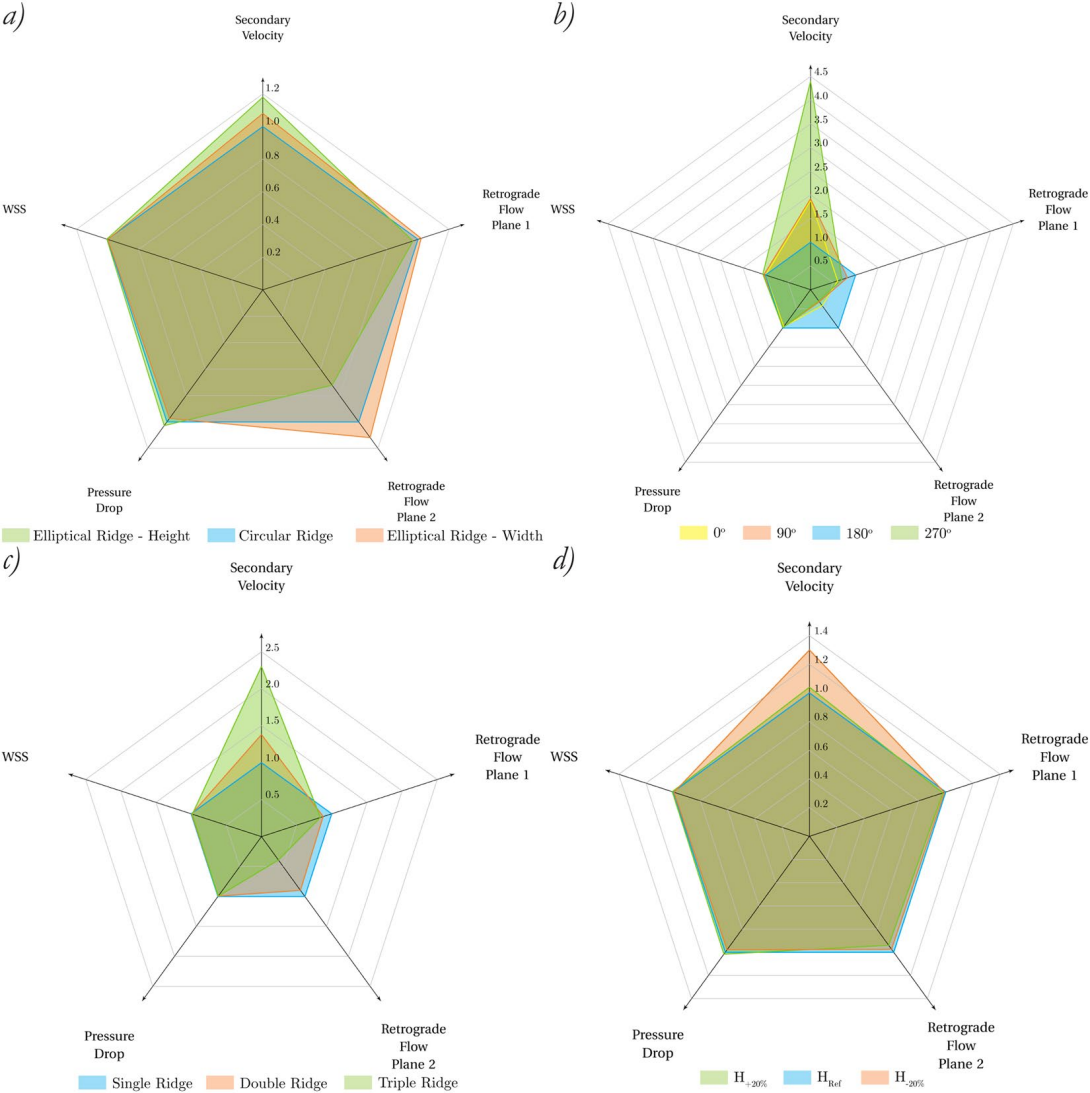
Overall comparison of normalised results for different ridge designs.

Figure 5 summarises the effects of different ridge designs on all the design parameters tested here, namely average secondary velocity magnitude at plane 3, area affected by retrograde flow, pressure drop and spatial mean of WSS. The results have been normalised by the corresponding values of the reference configuration.

Based on Fig. 5a, an improvement in the performance of the graft could be achieved through the variation of the height of an elliptical ridge. The increase of the ridge height has resulted in an enhancement of the swirling flow with higher values of secondary velocity, a reduction of the recirculation zones and a slight increase in the spatial mean WSS. These results are consistent with those obtained by Kabinejadian *et al*.^11^ using a different design strategy.

In the present study, the orientation of the spiral ridge trailing edge at the anastomosis has been shown to be the most important design parameter in order to improve the performance of the graft. As shown in Fig. 5b, significant differences in the spiral flow patterns and the regions of flow separation have been observed amongst the four trailing edge orientations tested here. In a lower order of magnitude, the variation of the ridge orientation also results in the most noteworthy difference in spatial mean of WSS in the host artery when compared with the other design parameters, producing increases of 3.442% and 5.969% with orientations 90° and 270°, respectively. The most significant effect of the trailing edge orientation was observed on the interaction between the induced rotation by the ridge and the suppression produced by the near sidewall of the host artery upon impingement. Consequently, in the case of the trailing edge orientation 270°, the intensity of the swirling flow is effectively enhanced greater than the other configurations due to the lesser degree of suppression from the surrounding boundaries, when passing through the anastomosis. These results are in agreement with those obtained by Keshmiri *et al*.^12^. The above observation has an important implication on the peripheral artery bypass surgeries, since the rotation of the blood flow has been found as clockwise in the left common iliac arteries and anticlockwise in the right common iliac arteries^34, 41^. Although the current design of SLF grafts incorporate the internal ridge with 180° orientation, the present findings could lead to a more effective peripheral bypass surgery strategy by considering two grafts with optimised ridge orientation and with opposite ridge rotational direction (i.e., clockwise and anticlockwise) in order to achieve the improved and physiologically suitable rotation of the blood flow depending on the location of the stenosis.

Most of the results presented in this paper including Fig. 5c suggest that increasing the number of ridge in the graft could have measurable effects, especially on the secondary velocity magnitude and retrograde flow. However, the impact of varying the number of ridges is more influenced by the location of the ridge trailing edge(s) as opposed to the number of ridges.

Figure 5d denotes a measurable improvement in terms of the secondary velocity magnitude in the region of the host artery away from the anastomosis by decreasing the helical pitch. However, other results reported throughout this study suggest that the variation of the pitch tends to have minor influences on the region of the anastomosis.

### Proposed Enhanced Design

Building on the results presented earlier, an enhanced design of spiral graft is proposed for its comparison against the conventional graft (no ridge) and spiral graft (configuration with single circular ridge and trailing edge orientation 180°). The new enhanced spiral graft is developed according to the most significant design parameters, i.e. single elliptical ridge (L_1_/D = 0.3) and orientation of the trailing edge 270°.

**Figure 6:**
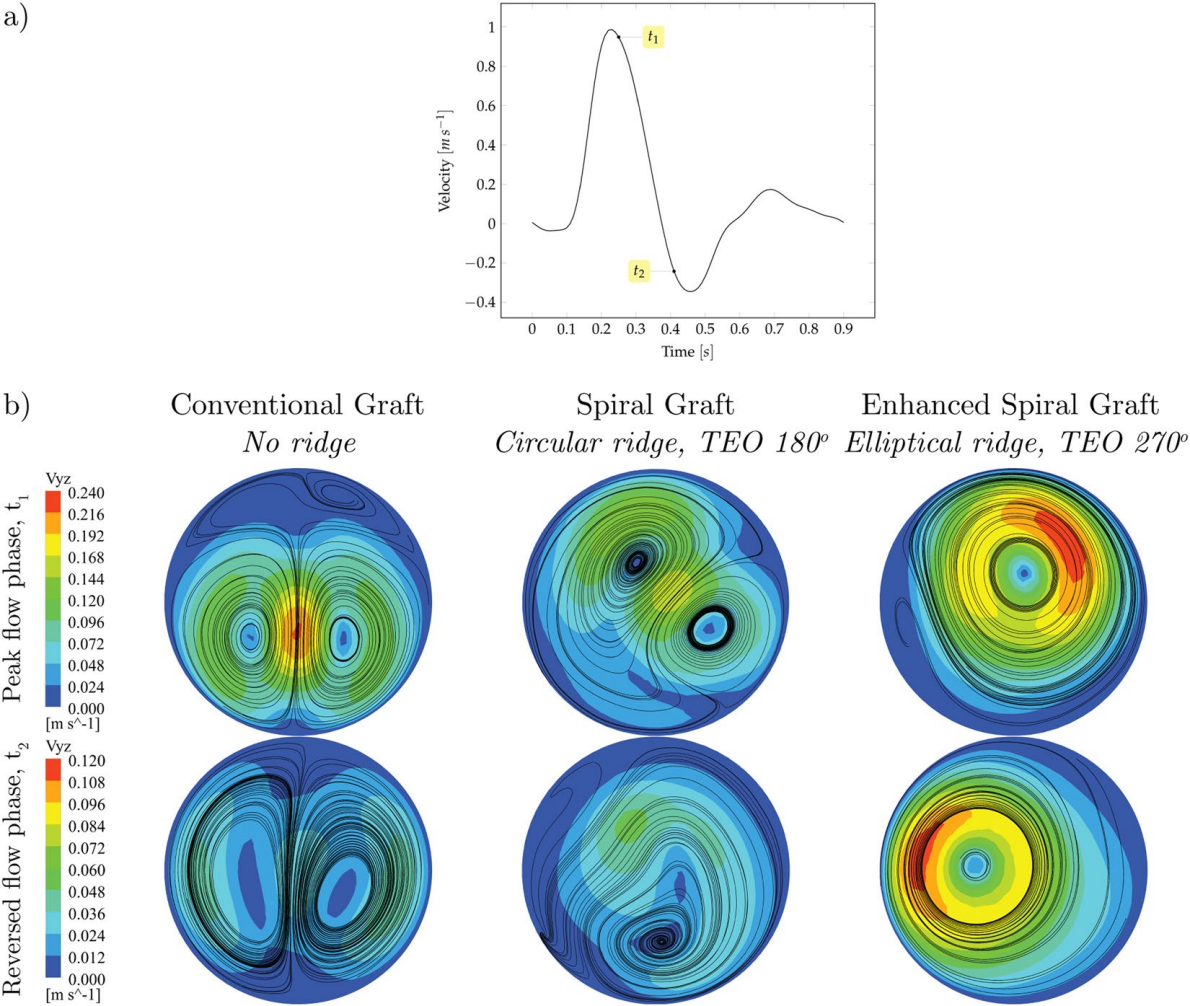
(**a**) Velocity waveform at the inlet and (**b**) contours of secondary velocity and crossflow streamlines at monitoring plane 3 and at peak (t_1_) and reversed (t_2_) flow phases.

The comparative analysis between conventional, spiral and enhanced spiral grafts has been carried out under transient boundary conditions using the inlet velocity waveform represented in Fig. 6a that corresponds to MRI measurements in the femoral artery of a healthy subject^42^ (details of the transient simulation can be found in Section “Computational Analysis”, above).

Figure 6b shows the contours of secondary velocity and crossflow streamlines at plane 3 and at peak (t_1_ = 0.25 s) and reversed (t_2_ = 0.41s) flow phases. The incorporation of the spiral ridge and its enhancement results in an increase of rotation and asymmetry of the transverse flow field. At both phases, the configuration of enhanced spiral graft shows a single dominant spiral and the complete development of the swirling flow, attributed to the dissipation of weaker vortices.

A decrease in area-weighted secondary velocity magnitude can be observed by comparing the conventional (0.067 m s^−1^) to spiral (0.061 m s^−1^) grafts at the peak flow phase. This slight discrepancy is caused by the ‘underdeveloped’ swirling flow in the spiral graft case. This observation is in agreement with the investigation by Bechara *et al*.^37^ where it was shown that the spiral flow graft did not lead to higher patency rates in comparison to conventional graft.

Transient haemodynamics metrics, such as Time-Averaged WSS (TAWSS), TAWSS Gradient (TAWSSG), Oscillatory Shear Index (OSI) and Relative Residence Time (RRT), have been identified as important fluid dynamic parameters to predict the development of cardiovascular diseases under physiological pulsatile conditions^11^. Such fluid dynamic variables are analysed according to the widely accepted low/oscillatory shear theory^28^.

**Figure 7:**
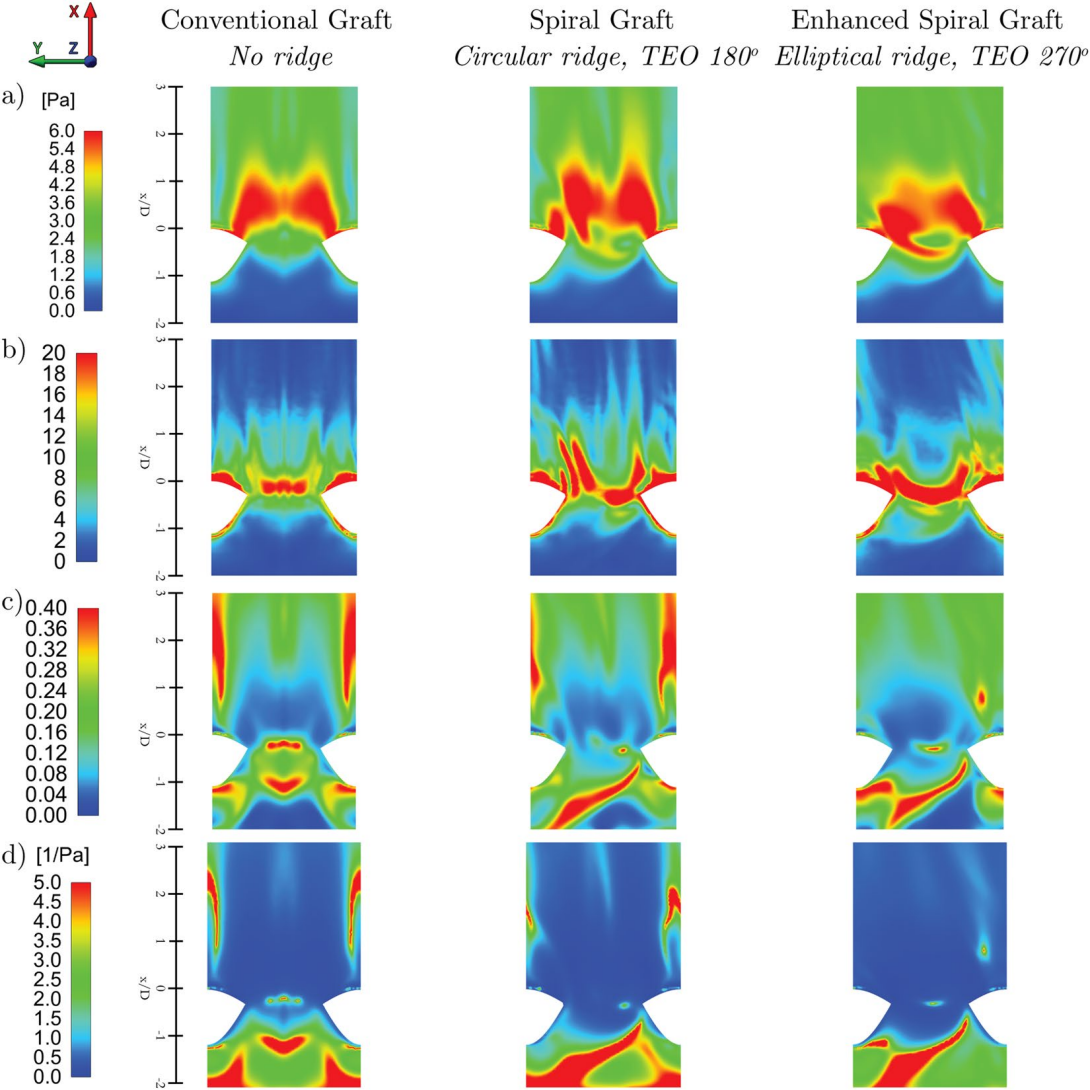
Distributions of (**a**) TAWSS, (**b**) TAWSSG, (**c**) OSI and (**d**) RRT onto the developed surface of the host artery for different designs of graft.

Figure 7a shows the distributions of TAWSS on the unfolded model of the host artery. TAWSS is defined through:

7 $$TAWSS=\frac{1}{T}{\int }_{0}^{T}|{\overrightarrow{{\boldsymbol{\tau }}}}_{w}|dt$$

where ${\overrightarrow{{\boldsymbol{\tau }}}}_{w}$ is the WSS vector and *T* is the period of the flow cycle corresponding to 0.9 s in the present case. An increase of the asymmetry intensity of the TAWSS is observed when the spiral ridge is added to the graft. The higher intensity of the swirling flow when the spiral ridge is enhanced, mainly caused by the orientation of the ridge, slightly extends the region of high-TAWSS towards the occluded section, as was observed under steady-state conditions.

Figure 7b shows the distributions of TAWSSG defined as:

8 $$TAWSSG=\frac{{D}_{G}}{{\tau }_{o}}\frac{1}{T}{\int }_{0}^{T}\sqrt{{(\frac{\partial {\tau }_{x}}{\partial x})}^{2}+{(\frac{\partial {\tau }_{y}}{\partial y})}^{2}+{(\frac{\partial {\tau }_{z}}{\partial z})}^{2}}dt$$

where *D*_*G*_ is the diameter of the graft (6 mm) and *τ*_*o*_ is the WSS corresponding to the Poiseuille flow under average flow conditions (0.82 Pa). Regions of high TAWSSG are identified in the anastomoses at the toe, heel and the stagnation point on the bed of the host artery. The spiral ridge shifts and extends these regions, while the enhanced spiral ridge generates a non-uniform band of high TAWSSG in the cross-sectional plane of the toe.

The analysis of OSI and RRT are shown in Fig. 7c and d and described in equations 9 and 10, respectively:

9 $$OSI=\frac{1}{2}(1-\frac{|{\int }_{0}^{T}{\overrightarrow{{\boldsymbol{\tau }}}}_{w}dt|}{{\int }_{0}^{T}|{\overrightarrow{{\boldsymbol{\tau }}}}_{w}|dt})$$

10 $$RRT=\frac{1}{(1-2\times OSI)\times TAWSS}=\frac{1}{\frac{1}{T}|{\int }_{0}^{T}{\overrightarrow{{\boldsymbol{\tau }}}}_{w}dt|}$$

Regions of high OSI and RRT are initially symmetrical and located at the heel (and around the impingement point in the case of conventional graft). The incorporation of the spiral ridge results in an oblique elevated region within the anastomosis and occluded region. The effect of the spiral ridge also results in asymmetry of the high OSI and RRT region distally extended from the toe which is dissipated by means of the enhancement of the ridge design.

Finally, it is worth noting that the similarities between the results obtained by the enhanced spiral graft and those obtained by Kabinejadian *et al*.^11^ using a non-planar (out-of-plane) helical graft in terms of TAWSSG, OSI and RRT. This once again highlights the potential benefits of using mixed design strategies by incorporating optimised geometries of out-of-plane helical graft and spiral ridge.

### Limitation and Future Direction

While the present study represents an important step towards improving the design and performance of spiral-inducing grafts, in order to find the ‘optimum’ configuration, further work is required to construct an appropriate ‘cost function’ against which to perform shape optimisation. The range of possible design configurations in the problem presented in this work is also large, therefore one would have to extend the number of design parameters and their range in order to undertake a complete shape optimisation. Furthermore, the interaction of the spiral ridge and out-of-plane grafts need to be investigated to match the direction of the spiral ridge with that resulting from the graft out-of-plane to maximise helicity. Moreover, further studies are required in order to test a wider range of physical, geometrical and hemodynamic properties of both helical and spiral designs with a view to be utilised in different types of bypass grafts. This is the subject of a future work for the present authors. Another possible future extension for the present study would be to study the proximal anastomosis, where the size of the ridge and the shape of the leading edge could lead to an increase in fluid resistance, the damage of inflow conditions to the prosthetic graft and the deposition of cholesterol, calcium, cellular debris and fatty substances at the leading edge.

## Conclusions

Cardiovascular diseases are one of the leading causes of death in the world. Arterial Bypass Grafts (ABGs) and Arterio-Venous Grafts (AVGs) have become a widely used treatment of blood vessel replacement and vascular access, respectively, in particular for high-risk patient. It is now widely accepted that unfavourable haemodynamic conditions play an essential role in the formation and development of intimal hyperplasia, which is the main cause of graft failure. The aim of the present study was to optimise the design of a novel bypass graft which incorporates an internal spiral ridge. This bypass graft would create a positive flow features and more favourable distribution of hemodynamic parameters in the graft, anastomotic region, and the host artery, which in turn, could enhance the patency and longevity of the bypass graft. In this paper, different design parameters were studied and it was found that the height of the elliptical ridge and the orientation of the trailing edge have the most important effects in enhancement of the graft design. These findings, together with the physiologically different direction of rotation of the flow depending on the artery, allows for consideration of a novel strategy for peripheral bypass surgery. Therefore, to achieve enhanced surgical outcome, the present results suggest that two grafts with symmetrically optimised trailing edge orientation and with opposite ridge rotational direction (i.e., clockwise and anticlockwise) should be available to the surgeon to choose from depending on the location of the stenosis. Finally, patient-specific waveform flow inlet was used to calculate advanced haemodynamics metrics, which revealed the potential haemodynamic benefits of the proposed enhanced design compared to the control and standard spiral graft.

## Electronic supplementary material

Supplementary Figures
