# Supplementary material for: Optimisation of a Novel Spiral-Inducing Bypass Graft Using Computational Fluid Dynamics
Source: Sci Rep. 2017 May 12;7:1865. doi: 10.1038/s41598-017-01930-x (PMC5431846; doi:10.1038/s41598-017-01930-x)
Supplement: Supplementary file 1 — Supplementary Figures [file 41598_2017_1930_MOESM1_ESM.pdf]

SUPPLEMENTARY DATA

Optimisation of a Novel Spiral-Inducing Bypass Graft Using Computational Fluid Dynamics

Andres Ruiz-Soler[1], Foad Kabinejadian[2], Mark A. Slevin[3], Paulo J. Bartolo[4], and Amir Keshmiri[4,*]

[1]*Engineering and Materials Research Centre, Manchester Metropolitan University, Manchester, M1 5GD, U.K.*

[2]*Department of Biomedical Engineering, University of Michigan, Ann Arbor, 48109-2110, USA*

[3]*Healthcare Science Research Centre, Manchester Metropolitan University, Manchester, M1 5GD, U.K.*

[4]*School of Mechanical, Aerospace and Civil Engineering (MACE), The University of Manchester, Manchester, M13 9PL, U.K.*

[*] *Corresponding author: email: a.keshmiri@manchester.ac.uk,*

Tel: +44 (0) 161 306 5752.

Figures

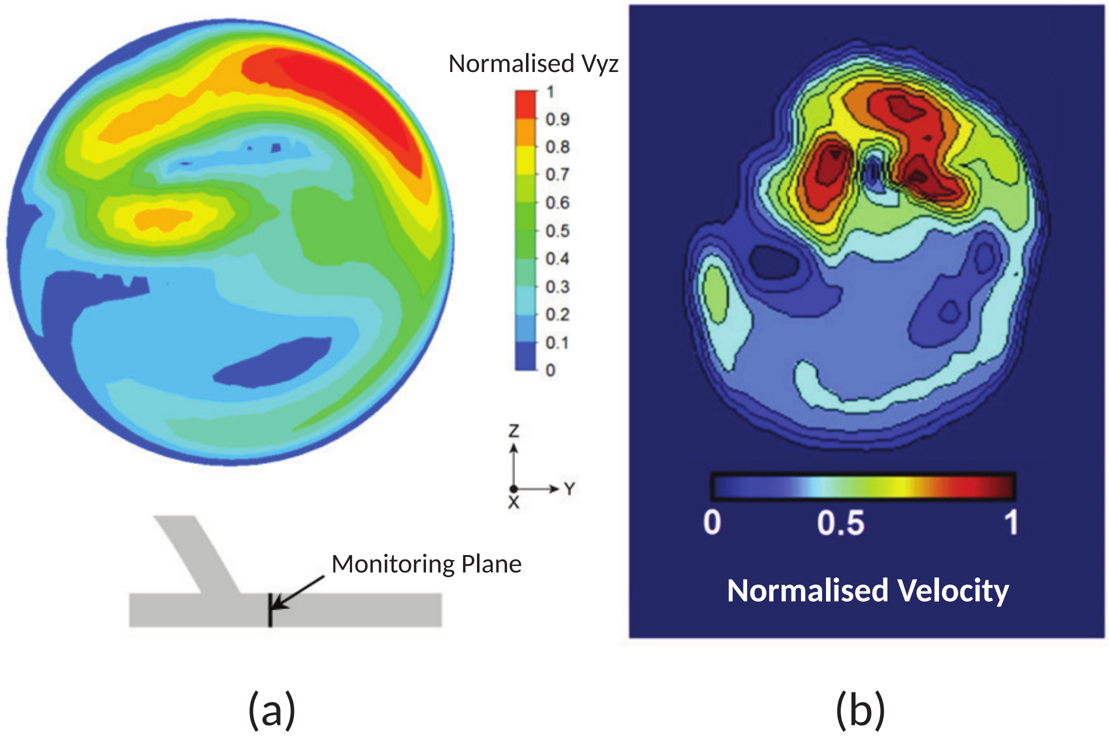

Supplementary Figure S1. Qualitative comparison of the normalised secondary velocity magnitude in the present reference model against the experimental data of [Kokkalis, E. et al. Secondary flow in peripheral vascular prosthetic grafts using vector Doppler imaging. Ultrasound Med. Biol. 39, 2295–307, 2013], obtained using dual-beam vector Doppler at a monitoring plane, positioned 5 mm distal from the graft outflow. The Reynolds number is set to *Re* = 1140 in this case. It can be seen that the numerical results are in good qualitative agreement with the data, especially in identifying the spiral flow in the host artery and areas with the maximum velocity magnitude.

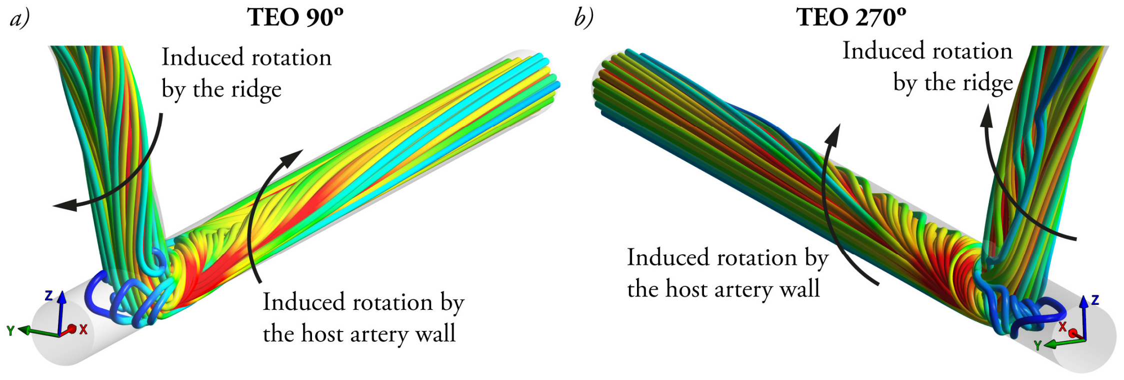

Supplementary Figure S2. Streamlines and representation of the rotation induced by a single ridge with trailing edge orientations 90º and 270º, and those produced by the curvature of the artery wall.

a)

	Double Ridge	
	$0°, 180°$	$90°, 270°$
$V_{yz}\ [m\,s^{-1}] \cdot 10^{-3}$, Plane 3	6.87	14.42

b)

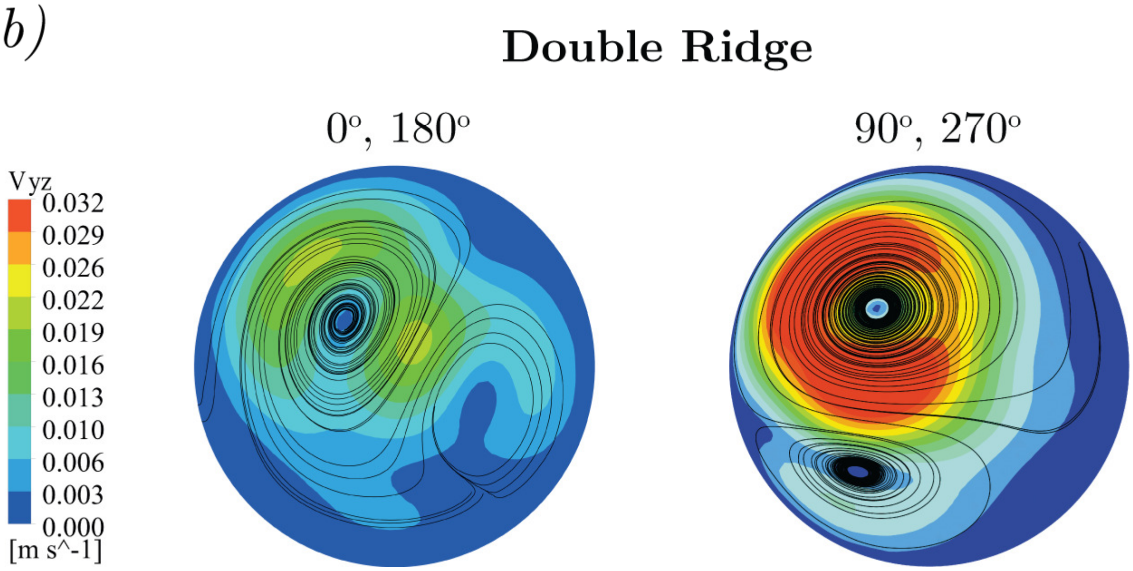

Supplementary Figure S3. a) Area-weighted average and b) contours of secondary velocity magnitude at monitoring plane 3 when comparing the double ridge configuration with trailing edge orientations 0º/180º and 90º/270º.
